# Balance assessment in HTLV-1 associated myelopathy or tropical spastic paraparesis

**DOI:** 10.1590/0037-8682-0388-2020

**Published:** 2020-11-25

**Authors:** Naiane Araújo Patrício, Mônica Andrade Rios, Patrícia Carvalho Barbosa, Jéssica Ramos Ribeiro, Diogo Guedes Vidal, Kátia Nunes Sá, Abrahão Fontes Baptista

**Affiliations:** 1 Universidade Federal da Bahia, Pós-graduação em Medicina e Saúde, Salvador, BA, Brasil.; 2 Escola Bahiana de Medicina e Saúde Pública, Graduação de Fisioterapia, Salvador, BA, Brasil.; 3 Universidade Fernando Pessoa, Unidade de Investigação UFP em Energia, Ambiente e Saúde (FP-ENAS), Porto, Portugal.; 4 Escola Bahiana de Medicina e Saúde Pública, Salvador, BA, Brasil.; 5 Universidade Federal do ABC, Centro de Matemática, Cognição e Computação, São Bernardo, SP, Brasil.; 6 Universidade de São Paulo, Laboratório de Investigações Médicas 54 (LIM-54), São Paulo, SP, Brazil.

**Keywords:** Postural balance, Tropical spastic paraparesis, Human T-lymphotropic virus 1, Health evaluation

## Abstract

**INTRODUCTION:**

A good rating of the device in people with HTLV-1 in this population is essential for accuracy in prescribing data (walking). Thus, this study aimed to analyze the counterpart assessment methods that are best suited to patients with human T-cell lymphotropic virus (HTLV)-1 associated myelopathy or tropical spastic paraparesis (HAM/TSP).

**METHODS:**

This cross-sectional study related stabilometric and kinematic variables of postural oscillations with Berg’s balance scale (BBS) and Timed Up and Go (TUG) in subjects with HAM/TSP compared to asymptomatic subjects. To assess the posterior and lateral postural projection, baropodometry and the Footwork® system was used, and the CVMob system was applied to kinematic parameters. The means comparison tests and correlations were applied with an alpha of 5%.

**RESULTS:**

Thirty-nine subjects (predominantly female) made up the sample. There was an increase in barodopometric oscillations, in the total oscillation area (p = 0.004), in the anteroposterior oscillation in the left (p = 0.015) and right views (p = 0.036), and in the lateral oscillation (p = 0.039) in the HAM/TSP group. Moderate correlations were found between oscillation baropodometry and the angular variation of the ankle, as well as with the BBS in the three angles and the TUG for lateral oscillation (p = 0.406).

**CONCLUSIONS:**

Each method has advantages and disadvantages, including cost accuracy. The best resources available at no additional cost for outpatient to use are the kinematic evaluation using a simple smartphone camera and free analysis software, and the TUG.

## INTRODUCTION

The human T-cell lymphotropic virus (HTLV) infects 10 to 20 million people on all continents, but Brazil is the country with the highest absolute number of known cases^1^. Although the majority of those infected remain asymptomatic, about 5%-10% of patients may progress to neoplastic, inflammatory, or degenerative diseases. HTLV-1 associated myelopathy or tropical spastic paraparesis (HAM/TSP) is very frequent in this population^2^. The posture of people with HAM/TSP is typically altered by spasticity, shortening, and weakness of muscle groups in the lower limbs^3^. This pattern affects gait^4^ and balance, causing frequent falls^5^ ^,^ ^6^.

Balance assessment is, thus, very important in this population, and should be done routinely in the clinical setting to identify potential risks of falls. This can be accomplished by the use of biomechanical measuring instruments or by scales and functional tests. A simple and accurate method is preferred for use in the clinical setting. Biomechanical methods are the gold standard for balance assessment and include the use of force platforms, baropodometry, dynamic posturography and kinematics, and multiaxial stabilometry^7^, most of which are applied in research laboratories with technically trained examiners. Among the available biomechanical tools, the ones that present the best cost-time-accuracy ratio to be adopted in clinical practice are baropodometric^8^ ^,^ ^9^ ^,^ ^10^and kinematic evaluations^3^ ^,^ ^11^. Both methods can be used to assess orthostatic posture by analyzing stabilometric and angular oscillation data.

Functional tests and scales are less accurate than biomechanical methods in identifying balance disturbances, but are easier to use in the clinical setting, as they can be used without technological tools or highly trained technical staff. Of these tests, the most popular are the Timed Up and Go (TUG) test, the Romberg Test, the Berg Balance Scale (BBS), and the Dynamic Gait Index^7^. BBS is the most commonly used in HAM/TSP^12^ ^,^ ^13^. For evidence-based clinical practice, however, it is necessary to select a more accurate, less costly, and briefer application method. Data obtained by a more accurate outpatient method may be favorably accepted by translational science^14^. Also, the systematic use of this method in the follow-up of infected individuals may allow for greater accuracy in balance training and the prescription of walking aids^13^. The present study aimed to analyze which of these balance assessment methods are best suited for people with HAM/TSP.

## METHODS

This cross-sectional study was conducted on subjects registered for research trials and specialized assistance in the city of Salvador, Bahia, Brazil. The study was approved by the Research Ethics Committee of the Catholic University of Salvador under CAAE 49634815.2.0000.5628, constituting a specific objective of a crossover clinical trial. All participants, after being informed of the collection objectives and procedures, signed the consent form that followed the recommendations of the Helsinki Declaration and National Health Council Resolution 466/12.

Data collection was carried out in the biomechanics laboratory of the Catholic University of Salvador, from April 2016 to January 2017, by a trained team of physiotherapists. The evaluated population was comprised of patients seropositive for HTLV-1, with defined or likely HAM/TSP, according to the guidelines of the World Health Organization (WHO) published in 1988, revised in 1989^15^, in addition to the ability to remain in orthostasis for at least 30 seconds. Non-inclusion criteria were cases of co-infection with human immunodeficiency virus or viral hepatitis, pregnancy, psychiatric disorders, rheumatic or orthopedic diseases, other neurological disorders according to the responsible neurologist, and those who had difficulties in understanding the assessment instruments and controls used.

Initially, a sociodemographic questionnaire was applied to obtain clinical information on the subjects’ history of falls. Then, participants, dressed in sports or intimate clothes, were positioned onto the baropodometry platform (FootWork Pro, AM cube®, Gargas, France), and were instructed to remain stable for 30 seconds with the head straight, eyes open and gaze fixed on a point marked on the wall to guarantee the horizontality of the plane of Frankfort. The baropodometer was calibrated with the weight (Welmy Precision Scale, Welmy, Salvador, Brazil) and stature (Cescorf® stadiometer, Salvador, Brazil) of the participant. For baropodometry evaluation, through the FootWork® software, we considered the total area of oscillation (TAO) in cm², and anteroposterior (APO) and lateral oscillations (LO) in cm. 

**FIGURE 1:**
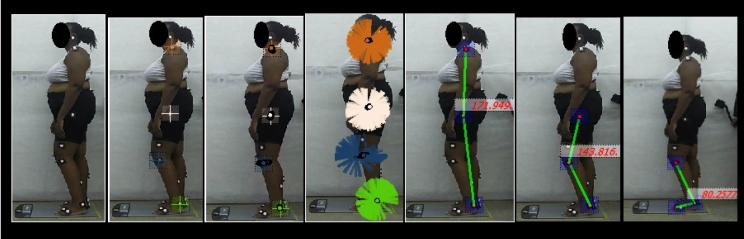
Kinematic points of the evaluation (body alignment, hip and ankle angles) of a participant with HAM/TSP, Salvador, Bahia, Brazil, 2017.

**FIGURE 2:**
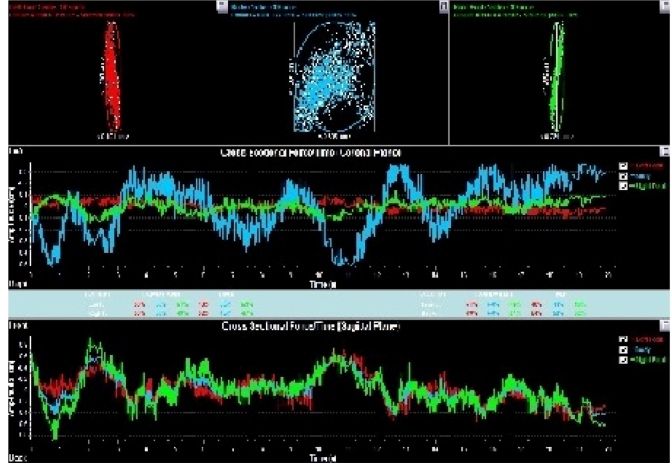
Stabilometric evaluation of a participant with HAM/TSP, Salvador, Bahia, Brazil, 2017. Legend: blue color - total area of oscillation of body; red and green color - anteroposterior and lateral oscillations of the feet).

Video recordings were performed during all the tests using a video camera (GoPro HERO 3.0, GoPro Inc.®, San Mateo, Califórnia, USA) in the sagittal, and left and right views. For the delimitation of the anatomical landmarks, the SAPO® Protocol (http://sapo.incubadora.fapesp.br/portal) was used. The anatomical points were marked using 25 mm semi-beads affixed with double-sided Scotch® adhesive tape. The video camera was adjusted using an Elgin adjustable height tripod (TEEM® TM 3180, Rio de Janeiro, Brazil). The tripod was positioned three meters from the platform and at half the height of the participant. Subsequently, the video was transferred to a laptop, and the kinematic evaluation was performed using CVMob. The kinematic variables analyzed were body alignment, and hip and ankle angles (Figure 1 and Figure 2).

After baropodometry, participants were assessed for balance using the BBS^16^ and then the TUG^13^ ^,^ ^17^. Briefly, the BBS consists of 14 activities, scored from 0 to 4, with a maximum value of 56 points. In the TUG, the participant was seated in a standard chair, approximately 46 cm away from the floor, and then stood up, walked a distance of 3 meters, returned to the chair and sat again. The test was performed once for training, and the result of the second attempt was recorded. The time was digitally monitored. The participants were assessed individually to guarantee privacy and exclusivity.

A sample size calculation was performed according to the online calculator of the Laboratory of Epidemiology and Statistics of the University of São Paulo (LEE), considering a standard deviation of 9.83 of the Berg Scale scores, a between-group difference in the BBS of 10 points^18^, a 5% alpha value, and study power of 80%. According to these characteristics, a sample size of 12 participants was estimated for two groups, one with HAM/TSP and the other composed of healthy participants. The comparative group was composed of an HTLV seronegative family member or caregiver, systematically assessed by a multidisciplinary team at the reference center. 

All data were tabulated and analyzed by the Statistical Package for the Social Sciences (SPSS®, IBM SPSS, University of Chicago, USA) version 25.0. Initially, descriptive analyses were performed through the distribution of absolute numbers and proportions for categorical variables, with mean and standard deviation for the normally distributed quantitative variables. The Shapiro-Wilk test was used to test normality. The sociodemographic characteristics of the two groups were compared using the chi-squared test. For continuous variables, the t-test for independent samples was used. For the inferential analyses of the differences in the measures, an unpaired t-test or Mann Whitney U test was used, according to the distribution. To test the correlation, the Spearman’s correlation test was applied. The construction of composite variables was based on the compute variable option in SPSS to create a new variable that results from the joining of two. The acceptable level of significance was 5%. 

## RESULTS

The sample consisted of 39 people, 26 in the HAM/TSP group and 13 in the comparative group. The mean age was 50.7 ± 9.8 years old in the affected group and 49.3 ± 9.4 in the accompanying group. Subjects were mostly female, Catholic, single and/or married and with self-reported brown skin color. In the sample, 14 (53.8%) people with HAM/TSP who used walking aids and 18 (69.2%) who did not, reported the occurrence of more than two falls in the last three months (Table 1). An average BBS score of 41.7 ± 7.8 and a TUG value of 20.5 ± 9.7 seconds with differences between groups were observed (p < 0.001). The stabilometric and postural variables were asymmetric between the groups. Differences between groups were observed in the total area of oscillation in the left lateral view (p = 0.004), in the anteroposterior oscillation on the left (p = 0.015) and right (p = 0.036), as well as in the lateral-lateral oscillation in the right lateral view (p = 0.039). The body alignment angle showed a statistically significant difference in the left (p = 0.002) and right (p < 0.001) views, as did the ankle angle in the left lateral view (p = 0.031) (Table 2).

**TABLE 1:** Sample sociodemographic characterization of individuals with and without HAM/TSP, Salvador, Bahia, Brazil, 2017.

	HAM/TSP Group 26		Comparative group 13	
	N	%	N	%
**Sex**				
Male	9	34.6	3	23.1
Female	17	65.4	10	76.9
**Marital Status**				
Single	12	46.2	2	15.4
Marriage	10	38.5	9	69.2
Divorced	0	0.0	1	7.7
Widower	4	15.4	1	7.7
**Skin Color**				
Yellow	1	3.8	0	0.0
White	1	3.8	2	15.4
Red	0	0.0	1	7.7
Brown	12	46.2	7	53.8
Black	12	46.2	3	23.1
**Physiotherapy**				
Yes	22	84.6	9	69.2
No	4	15.4	4	30.8
**Physical Activity**				
Yes	9	34.6	8	61.5
No	17	65.4	5	38.5
**Walking Aids**				
Yes	14	53.8	0	0.0
No	12	46.2	13	100
**Lateral Domain**				
Hight-handed	23	88.5	13	100
Left-handed	3	11.5	0	0.0
**Falls (last 3 months)**				
None	3	11.5	8	61.5
One	4	15.4	2	15.4
Two	1	3.8	1	7.7
More than two	18	69.2	2	15.4

**HAM/TSP:** HTLV-1 associated myelopathy or tropical spastic paraparesis.

**TABLE 2:** Demographic and functional characterization, stabilometrics, and kinemetric oscillations of individuals with and without HAM/TSP, Salvador, Bahia, Brazil, 2017.

		HAM/TSP		Comparative		
		Group 26		group 13		
		Mean (SD)		Mean (SD)		*p*
Age		50.7 ± 9.8		49.3 ± 9.4		0.659
**BMI**		23.7 ± 6.9		25.8 ± 5.2		0.246
**Berg Scale**		41.7 ± 7.8		55 ± 1.5		**<0.001**
**Timed Up and Go**		20.5 ± 9.7		8.66 ± 1.3		**<0.001**
**Left Lateral View Stabilometrics Oscillation**						
Total Area of Oscillations		5.95 ± 7.32		1.31 ± 1.09		0.004**
Antero-posterior Oscillations		2.65±1.10		1.74 ± 0.78		0.015*
Lateral Oscillations		2.48 ± 2.81		0.86 ± 0.44		0.057
**Right Lateral View Stabilometrics Oscillation**						
Total Area of Oscillations		4.85 ± 8.13		1.26 ± 1.47		0.140
Antero-posterior Oscillations		2.43 ± 1.23		1.58 ± 0.79		0.036*
Lateral Oscillations		1.91 ± 1.70		0.84 ± 0.47		0.039*
**Angular Kinemetric Left Lateral Oscillation**						
Body Alignment		1.05 ± 0.81		0.12 ± 0.79		0.002**
Hip Angle		168.1 ± 34.4		175.8 ± 3.65		0.446
Ankle Angle		83.2 ± 5.86		87.3 ± 2.92		0.031*
**Angular Kinemetric Right Lateral Oscillation**						
Body Alignment		1.28 ± 0.89		1.24 ± 0.49		0.000**
Hip Angle		167.8 ± 34.3		173.4 ± 3.75		0.580
Ankle Angle		95.9 ± 5.05		92.8 ± 2.84		0.052

**HAM/TSP:** HTLV-1 associated myelopathy or tropical spastic paraparesis; **BMI:** Body Mass Index; **SD:** Standard Deviation. *Statistical differences at 0.05 level and **Statistical differences at 0.001 level.

By correlating the baropodometry oscillations and the postural kinematics with the BBS scores, we identified statistically significant differences between the groups, with negative, weak to moderate correlations between the baropodometry oscillations (left lateral view) and the BBS on the left. The HAM/TSP group presented two moderate negative correlations compared to the group without HAM/TSP, in the anteroposterior oscillation in both views. No correlations were found between the postural oscillations and the BBS in both groups. By correlating baropodometry and postural kinematic oscillations with TUG scores, we identified statistically significant differences between the groups, with positive and moderate correlations between lateral stabilometric oscillation (left lateral view) and TUG in the group with HAM/TSP and moderately strong correlations in the body kinematic oscillation (right lateral view) in both views in the group without HAM/TSP (Table 3). 

**TABLE 3:** Correlation between baropodometry and kinemetric oscillations with the Berg Balance Scale in individuals with and without HAM/TSP, Salvador, Bahia, Brazil, 2017.

		Baropodometry Left View			Baropodometry Right View			Kinemetry Left View			Kinemetry Right View		
		TAO	APO	LO	TAO	APO	LO	Body Alignment	Hip Â	Ankle Â	Body Alignment	Hip Â	Ankle Â
BBS	**With HAM/TSP**	-0.505**	-0.399*	-0.495*	-0.291	-0.380	-0.225	-0.268	-0.228	0.359	0.007	0.069	0.005
	**Without HAM/TSP**	-0.334	-0.642*	-0.124	-0.334	-0.556*	-0.334	0.173	-0.025	0.000	-0.531	0.099	-0.420
													
TUG	**With HAM/TSP**	0.360	0.176	0.406*	0.259	0.313	0.234	0.183	0.256	-0.238	0.126	0.023	-0.044
	**Without HAM/TSP**	0.072	0.426	-0.041	0.234	0.283	0.292	-0.239	0.261	0.138	**0.644** ^*^	-0.184	0.074

**BBS:** Berg balance scale; **TUG:** Timed up and go test; **HAM/TSP:** HTLV-1 associated myelopathy or tropical spastic paraparesis; **Â:** angle; **TAO:** Total area of oscillation; **APO:** Anteroposterior oscillation; **LO:** Lateral oscillation. Spearman correlation (*Correlation significant at 0.05 level and **Correlation significant at 0.001 level)..

To verify the existence of a relationship between the different assessment methods, two composite variables were created, i.e. TUG plus kinemetric data and BBS plus stabilometric data. The composite TUG plus kinemetry was correlated with BBS and each one of the stabilometric variables (Table 4).

**TABLE 4:** Correlation between TUG plus kinemetric data composite variable and BBS, stabilometric and BBS plus kinemetric data composite variables in individuals with and without HTLV-1, Salvador, Bahia, Brazil, 2017.

	**TUG *plus* Kinemetry**	**TUG *plus* Kinemetry**
	HAM/TSP Group	Comparative Group
	N = 39	N = 26
BBS *plus* stabilometry	-0.439*	-0.374
BBS	-0.728**	-0.581**
Total Oscillation Area (Front View)	0.303	-0.130
Total Oscillation Area (Back View)	0.291	-0.024
Anteroposterior Oscillation (Front View)	0.204	-0.051
Anteroposterior Oscillation (Back View)	0.365*	0.094
Lateral Oscillations (Front View)	0.356*	0.211
Lateral Oscillations (Back View)	0.267	-0.042

**BBS:** Berg balance scale; **TUG:** Timed up and go test; **HAM/TSP:** HTLV-1 associated myelopathy or tropical spastic paraparesis. *Correlation significant at 0.05 level and **Correlation significant at 0.001 level.

## DISCUSSION

This study aimed to describe the postural control examination responses in people with HAM/TSP to analyze which of the balance assessment methods are best suited to this population. The results indicate that people living with this neurological disease have greater center of gravity oscillations compared to uninfected individuals, characterized by both increased stabilometric oscillations and body and ankle kinematic angles. 

Due to several reports of falls in the sample, people with HAM/TSP were expected to use among the main strategies to keep the body in balance on the support base, the hip strategy, since the ankle strategy is generally insufficient in populations with a high risk of falling^19^. However, no large variations were observed in hip angle kinematics. This finding can be explained by good motor control of the pelvic stabilizing muscles, since many participants have previously practiced or currently practice Pilates exercises^20^ ^,^ ^21^. This sample also usually works on stretching and strengthening plantar flexors, which may increase the efficiency of balance control by the ankle strategy^22^.

Examination of all methods confirmed that this is a population with balance deficits that produces a total oscillation area expansion six times greater than that of uninfected persons. This finding justifies the high occurrence of falls reported by participants, which is in agreement with previous studies^23^ ^,^ ^24^. Muscle weakness and lower limb spasticity are associated with postural instability^3^ ^,^ ^25^and reduced functional mobility^13^ ^,^ ^23^. Sensory disturbances of the central nervous system with inadequate lower limb muscle function generate postural instability^21^ and, consequently, an increased risk of falls.

Postural sway is considered the basis of the feedback system for recalibration of the center of gravity postural control system and is widely used as a clinical measure of balance capacity^26^. In the HAM/TSP population, a high standard deviation was observed in the stabilometric variables. This finding may express the different stages of disease evolution among the subjects^5^. Among the methods employed, the only one that was able to measure this high dispersion was the baropodometry platform, which has been identified as a valid and reliable resource in cross-sectional balance studies^10^. However, the strong correlation between stabilometric and kinemetric data observed in the present study suggests that the use of angular measurements produce similar results to those of baropodometry when applied in clinical practice.

A three-dimensional kinematic system can also measure oscillations in the three planes^27^. However, free software was not found for this analysis, and its use in the research environment is too costly to be adopted as the gold standard on an outpatient basis. Thus, two-dimensional kinematics may be useful in the evaluation of this population^11^, but with restrictions in the data analysis. Despite the limitation of performing only two-dimensional analyses, the CVMob software used in the present study for kinematic analyses, proved useful in the evaluation of stabilometric oscillations at the three selected angles, with moderate correlation with the BBS and with the TUG, regarding lateral oscillation. These findings point to the indication of the use of kinematics with the aid of simple images acquired with smartphone cameras in the follow-up of these patients. With an application time (about 60 seconds for calibration and collection) less than or equal to the scales (30 minutes for BBS and 60 seconds for TUG) and baropodometry (about 90 seconds), it was able to detect the general equilibrium state. Measurements of angular variations, especially the ankle, may serve as a marker of balance based on the ankle strategy according to the ‘inverted pendulum’ theory^28^ ^,^ ^29^.

Stabilometry is the most accurate exam due to its ability to perform three-dimensional measurements and analysis, but its cost is around $5,000, which makes it impossible to adopt on an outpatient basis, especially for public services in underdeveloped or developing countries and in the time of a world economic crisis. It is worth remembering that HAM/TSP is a condition that affects less favored populations from the socioeconomic point of view^1^ ^,^ ^30^.

The BBS is the most detailed exam that includes several tasks, but the examiner’s verbal commands greatly influences the behavior of the subjects. Besides, the interpretation of the data is subjective and it has a prolonged application time, which can lead to fatigue of the evaluator as well as anxiety in the subject due to the challenge of balancing and referring to previous losses, and also an increase in the cost due to the time spent with a specialized professional. The suggested cutoff point for the sample in this exam is 50 points^13^, for those who prefer to adopt it. In which case, it is recommended that the evaluator should be trained and that the same examiner should always apply the scale to compare balance before and after an intervention, to monitor clinical progress, and to prescribe walking aids. Due to the aforementioned limitations, a more objective and quick application measure has become necessary, so the use of short films can be considered to assess the balance of this population since the scores obtained are very close to those obtained by the BBS scale.

On the other hand, the TUG is a quick exam. However, it also depends on the experience of the examiner who guides the subject during data collection. The use of a stopwatch in this test adopts a less subjective variable, which reinforces its value in the dynamic balance examination^17^. Because it does not require a double task, which would increase the risk of falling during the test, it is an accurate and safe balance examination for various populations. A previous study suggested a cutoff point of 12.28 seconds for the HAM/TSP population^13^. The TUG added to the kinematic analysis can complement balance information with greater precision in the follow up of this population.

The TUG plus kinemetric composite variable presented a strong correlation with BBS and BBS plus stabilometric data. This shows that both clinical and biomechanical assessments give the same information and can to be acceptable by clinicians and researchers. The simple smartphone camera kinematics in the lateral view focused on the ankle and the TUG test were complementary measures to assess balance in people with HAM/TSP.

Among the limitations of the present study, it is admitted that the stage of the disease in the sample was not evaluated. If the sample had been more homogeneous, the dispersion measures might not be so high. However, because this is a rare disease with associated sensory disturbances, forming homogeneous samples is very difficult. Nor was the most accurate contemporary examination used for this assessment, which would be a multiaxial force platform for balance examination. However, its high cost and the unavailability of the equipment in the state of Bahia made its application unfeasible. Another aspect that could complete the balance analysis would be electromyography, which was not used in the present study.

We conclude that the HAM/TSP population has a high balance deficit that can be measured by scales and stabilometric and kinematic measurements. Although all forms of evaluation have advantages and disadvantages, varying costs, and different collection times, our findings demonstrate that simple smartphone camera kinematics in the lateral view focused on the ankle and the TUG test were complementary and the best suited measures to assess balance in people with HAM/TSP.
